# Differential Association of Geographical Region of Birth With Dementia Risk Across Black Women and Men

**DOI:** 10.1093/geroni/igaa057.2694

**Published:** 2020-12-16

**Authors:** Justina Avila-Rieger, Audrey Murchland, Nika Seblova, Maria Glymour, Adam Brickman, Nicole Schupf, Richard Mayeux, Jennifer Manly

**Affiliations:** 1 University of New Mexico, New York, New York, United States; 2 University of California, San Francisco, San Francisco, California, United States; 3 Columbia University Medical Center, New York, New York, United States; 4 University of California, San Francisco, California, United States; 5 Columbia University, New York, New York, United States

## Abstract

Risk of dementia is both racially and spatially patterned. Less is known about sex/gender differences in pathways linking birth place to late-life cognitive outcomes in older non-Latino Blacks. The 1464 Black men and women included in these analyses were Northern Manhattan residents. Cox regressions revealed that Stroke-Belt South (SB) and Non-Stroke-Belt South (NSB) birth was associated with a higher dementia risk, adjusted for birth year, childhood SES, and risk of death. Compared to Northern-born (NB) men, SB men had the highest risk, followed by NSB women and SB women, while NSB men and NB women had a similar risk to NB men. The higher risk for SB men and NSB women remained after adjusting for education, adult income, and CVD burden. Future work should identify why birth in the SB is uniquely detrimental for cognitive health among Black men, while birth in NSB has the strongest impact on Black women.

